# Development of a rapid and sensitive real-time diagnostic assay to detect and quantify *Aphanomyces invadans*, the causative agent of epizootic ulcerative syndrome

**DOI:** 10.1371/journal.pone.0286553

**Published:** 2023-06-15

**Authors:** Diem Tho Ho, Nameun Kim, Yoonhang Lee, Dongbin Yun, MinJi Sung, El-Matbouli Mansour, P. K. Pradhan, Neeraj Sood, Wi-Sik Kim, Chan-Il Park, Ki Hong Kim, Do-Hyung Kim

**Affiliations:** 1 Department of Aquatic Life Medicine, Pukyong National University, Busan, Republic of Korea; 2 PCR Reagent Development Group, Bioneer Corporation, Daejeon, Republic of Korea; 3 Clinical Division of Fish Medicine, University of Veterinary Medicine, Vienna, Austria; 4 Fish Health Management and Exotics, ICAR-National Bureau of Fish Genetic Resources, Lucknow, Uttar Pradesh, India; 5 Department of Aqualife Medicine, Chonnam National University, Yeosu, Republic of Korea; 6 Department of Marine Biology & Aquaculture, Gyeongsang National University, Tongyeong, Republic of Korea; Benha University, EGYPT

## Abstract

The oomycete *Aphanomyces invadans* causes epizootic ulcerative syndrome (EUS), a World Organization for Animal Health (WOAH)-listed disease that has seriously impacted a wide range of fish worldwide. Currently, only three conventional polymerase chain reaction (PCR) assays are recommended for the detection of *A*. *invadans*. The robust quantitative PCR (qPCR) assay has recently become more important due to its highly accurate nature and the applicability of qPCR-based environmental DNA (eDNA) detection in the monitoring of pathogens in aquatic environments. Therefore, in this study, we developed a novel TaqMan probe-based qPCR method to sensitively and quantitatively detect *A*. *invadans*. The assay limit of detection was determined using 10-fold serial dilutions of linearized *A*. *invadans* plasmid. Assay sensitivity was assessed in the presence of interfering substances and compared to three WOAH-listed primers using the mycelia and zoospores of *A*. *invadans* with and without fish muscle tissue. The assay specificity was also theoretically and experimentally assessed against other oomycetes, fish muscle tissue, and water samples. The assay’s repeatability and reproducibility were determined. In this study, the limit of detection of the developed assay was 7.24 copies of *A*. *invadans* genomic DNA per reaction (95% confidence interval (CI): 2.75 to 19.05 copies/reaction). The assay showed the same sensitivity in the presence of other substances. Compared to the WOAH-recommended PCR assays, this assay had 10-times higher sensitivity for all tested samples. There were no cross-reactions with other closely related oomycetes, fish muscle, or water samples, indicating that the assay was highly specific for *A*. *invadans*. The repeatability and reproducibility tests showed little variation, ranging from 0.1–0.9% and 0.04–1.1%, respectively, indicating the high consistency, repeatability, and reliability of the developed assay. This highly rapid, sensitive, specific, and consistent EUS qPCR assay would be of importance in transboundary disease management and the monitoring of pathogens in aquatic environments.

## Introduction

*Aphanomyces invadans*, a member of the organisms formerly known as water molds, is a fungus-like organism belonging to the Chromista group [1]. It causes epizootic ulcerative syndrome (EUS), one of the most destructive diseases affecting more than 160 wild and farmed freshwater and estuarine fish species [2] and continuously infecting new fish species [3–5]. Infection with *A*. *invadans* was first recorded in Japan in 1971 and has been reported in more than 26 countries across the North American, southern African, Asian, and Australian continents since then [4, 6]. Recently, infection with *A*. *invadans* was reported in West Virginia, a southeastern region of the United States [5]. The recent expansion of EUS to new regions and its ability to continuously infect new fish species pose a potential threat to the aquaculture industry.

The World Organization for Animal Health (WOAH) recommends several approaches for diagnosing *A*. *invadans*, including antibody-based, fluorescent peptide nucleic acid *in situ* hybridization, and conventional polymerase chain reaction (PCR) assays [7]. However, these methods were reported to have low specificity and low sensitivity, and are time-consuming [8, 9]. Real-time quantitative PCR (qPCR) is becoming the method of choice as it ensures a high degree of specificity by combining gene-specific primers and a qPCR probe, as well as extremely rapid and highly sensitive quantification [10]. Thus, the use of qPCR-based environmental DNA (eDNA) detection to monitor of pathogens in aquatic environments has rapidly attracted attention [11, 12]. However, such a method is not available for the detection and quantification of *A*. *invadans*. Therefore, we developed a TaqMan probe-based real-time PCR EUS assay to detect and quantify *A*. *invadans*, providing a rapid and sensitive tool with the potential to increase the accuracy of *A*. *invadans* diagnosis in routine and reference laboratories.

## Materials and methods

### Ethics statement

This study was approved by the Ethics Committee of Pukyong National University (approval number: PKNUIACUC-2021-11) and was conducted according to the Bioethics and Safety Act of the South Korean Ministry of Health and Welfare.

### *Aphanomyces invadans*-specific primer and probe design

The sequence of the hypothetical protein encoding gene of *A*. *invadans* isolates NJM0002 (Genbank accession number: QUSY01000001.1), and NJM9701 (Genbank accession number: XM_008868928) was obtained from the Genbank nucleotide database of the National Center for Biotechnology Information. A BLASTn search was conducted for this hypothetical protein-encoding gene in the NCBI nt database, and the sequence was aligned with the homologous sequences of closely related species using Megalign (DNAstar, Lasergene). The regions that were unique to only *A*. *invadans* and the tobacco mosaic virus (TMV) movement protein-encoding gene sequence (AY300161.1) were screened as potential primers and hydrolysis probes for *A*. *invadans* and the internal positive control (IPC), respectively, using Primer3Plus [13]. The amplification efficiencies of the potential primers were predicted using the pcrEfficiency web interface (http://srvgen.upct.es/efficiency.html) [14]. Simulate-PCR (http://sourceforge.net/projects/simulatepcr) [11] was employed to check the desired target amplification and non-target cross-reactions against a panel of oomycetes retrieved from the EMBL/Genbank sequence databases. The hydrolysis probe for *A*. *invadans* was labeled with 6-carboxy-fluorescein dye (FAM) as a reporter and an internal-excellent Bioneer Quencher (i-EBQ) as an internal quencher. The 3’ end of the probe was blocked with phosphate. The hydrolysis probe for IPC was labeled with pentamethine cyanine (Cy5) as the reporter and an internal-excellent Bioneer Quencher as an internal quencher. The 3’ end of the probe was also blocked with phosphate.

### Positive control plasmid construction

The *Aphanomyces invadans* strains NJM7901 and S1 used in this study were kindly provided by Professor El-Matbouli Mansour (University of Veterinary Medicine, Austria) and Dr. P. K. Pradhan (ICAR-National Bureau of Fish Genetic Resources, India), respectively. The genomic DNA of *A*. *invadans* NJM9701 was extracted using an AccuPrep^®^ Genomic DNA Extraction Assay (Bioneer, Korea) according to the manufacturer’s protocol. The genomic DNA of *A*. *invadans* was amplified using EUS-Hypo-F and EUS-Hypo-R primers (Table 1), subjected to electrophoresis, and the amplicons were purified using the AccuPrep^®^ PCR/Gel Purification Assay (Bioneer, Korea). The purified PCR product was inserted into a pBHA-T vector (pGEM^®^-T and pGEM^®^-T Easy Vector Systems, USA) using the AccuRapid^™^ TA Cloning Assay (Bioneer, Korea) and transformed into *Escherichia coli* DH5α cells. The plasmid was then purified from the transformed *E*. *coli* using the AccuPrep^®^ Plasmid Mini Extraction Assay (Bioneer, Korea). Clones were sequenced to check their similarity. Plasmid DNA was quantified using a Nanodrop One (Thermo Fisher Scientific, USA) and extrapolated into copy numbers according to a previous study [15].

**Table 1:** Primers used in this study.

Assays	Sequence (5’-3’)	Product size	Target gene	References
**Quantitative PCR**				
EUS-Hypo-F	ATTCCGCTGTGGACATGCTT	80	Hypothetical protein	This study
EUS-Hypo-R	CGAGATCGATCGCACGTGAA			
EUS-Hypo-P	[FAM]AGCAGTGCCGAT[i-EBQ] TGAGAACTCCGT[Phosphate]			
IPC-F	TGATAGTGGATACGTCTGTTTAGC	121	Movement protein	This study
IPC-R	CTCGTCGGCTCTTTCCATC			
IPC-P	[Cyanine5]ACCAGACACA[i-EBQ] CGCTCACACCTCCC[Phosphate]			
**Conventional PCR**				
Ainvad-2F	TCATTGTGAGTGAAACGGTG	234	Small subunit and ITS1 region	[20]
Ainvad-ITSR1	GGCTAAGGTTTCAGTATGTAG			
BO73	CTTGTGCTGAGCTCACACTC	564	ITS1, 5.8 ribosomal DNA, and ITS2 region	[9]
BO639	ACACCAGATTACACTATCTC			
ITS11	GCCGAAGTTTCGCAAGAAAC	550	ITS1 and ITS2 region	[19]
ITS23	CGTATAGACACAAGCACACCA			
18S-F	ACCTGGTTGATCCTGCCAG	2000	18S ribosomal RNA gene	[17]
18S-R	TGATCCTTCYGCAGGTTCAC			
ITS1	TCCGTAGGTGAACCTGCGG	800	18S ribosomal RNA gene, ITS1, 5.8 ribosomal DNA, ITS2 region, and 28S ribosomal DNA	[18]
ITS4	TCCTCCGCTTATTGATATGC			

### Real-time quantitative PCR assay

The EUS qPCR assay was performed using an Exicyler^™^ 96 Real-Time Quantitative Thermal Block (Bioneer, Korea). Each qPCR assay was conducted in a 25-μL reaction mixture containing 5 μL of DNA template, 600 and 400 nM of each primer and probe targeting the hypothetical protein-encoding gene and the internal positive control, respectively, 1X TaqMan probe, and 15 μL of 2· dual-Hotstart RT-qPCR master mix (Bioneer, Korea). After pre-denaturation at 95°C for 5 min, amplification was conducted with 45 cycles at 95°C for 5 s (denaturation) and 55°C for 5 s (annealing and extension).

### Analytical specificity test

#### Cross-reactivity test

The specificity of the EUS qPCR assay was subsequently validated with direct experimental evidence. The cross-reactivity of the EUS qPCR assay and three conventional PCR assays recommended by the WOAH (Table 1) were tested against DNA from a panel of oomycetes, healthy fish muscle samples, and water samples. Muscle samples were aseptically collected from muscle tissue located under the dorsal fin of four freshwater fish species, including common carp (*Cyprinus carpio*), dwarf gourami (*Colisa lalia*), snakehead (*Channa argus*), and catfish (*Silurus asotus*), which were purchased from commercial fish farms and/or aquarium in South Korea. Water samples were collected from a tank populated with healthy catfish that had not experienced an outbreak of EUS, as well as from the Nakdong River in Busan, South Korea (Table 2). River water samples were collected 1 m under the water surface at three different sites (each site was 5 km from another site). One hundred milliliters of fish-rearing water and one litter of Nakdong River water samples were filtered through 0.45-μm MCE Membrane filters (MF-Millipore^™^, Germany) as described in a previous study [16]. Filters were used to extract DNA. Oomycete and water sample DNA was extracted using the DNeasy Power Soil Pro Kit (Qiagen, Germany), and fish tissue DNA was extracted using the Dneasy Blood & Tissue Kit (Qiagen, Germany) according to the manufacturer’s protocol. Each test was performed in triplicate using the same PCR conditions as stated above. All DNA samples used in this study were amplified using 18S-F/ 18S-R primers [17] (Table 1) to confirm of amplifiable template DNA. Water samples were also amplified using ITS1/ITS4 primers [18] to confirm the presence of other oomycetes.

**Table 2:** Cross-reactivity test results.

Species	EUS qPCR assay		Conventional PCR assays				
	*A*. *invadans* signal	IPC signal	BO73/BO639	Ainvad-2F/Ainvad-ITSR1	ITS11/ITS23	ITS1/ ITS4	18S-F/ 18S-R
**Oomycetes**							
*Aphanomyces invadans* NJM9701	+	+	+	+	+	+	+
*Aphanomyces invadans* S1	+	+	+	+	+	+	+
*Aphanomyces sp*. KACC 43829	-	+	-	-	+	+	+
*Aphanomyces laevis* CBS478.71	-	+	-	-	+	+	+
*Aphanomyces frigidophilus* NJM9665	-	+	-	+	+	+	+
*Apiotrichum loubieri*	-	+	-	-	+	+	+
*Fusarium keratoplasticum*	-	+	-	-	+	+	+
*Antrodiella zonata*	-	+	-	+	+	+	+
**Fish**							
Common carp (*Cyprinus carpio*)	-	+	-	-	-	-	+
Dwarf gourami (*Colisa lalia*)	-	+	-	-	-	-	+
Snakehead (*Channa argus*)	-	+	-	-	-	-	+
Catfish (*Silurus asotus*)	-	+	-	-	-	-	+
**Water**							
Fish-rearing water	-	+	-	-	-	-	+
Nakdong river water	-	+	-	-	+	+	+

(-) Non-amplification; (+) amplification; IPC: internal positive control

### Analytical sensitivity

#### Limit of detection and cut-off value determination

The analytical sensitivity of the EUS qPCR assay was evaluated using standard curves generated by the amplification of positive control plasmid DNA. Standard curves were constructed using 3-fold serially diluted positive control plasmid DNA, including 1000, 333, 111, 37, 12.3, and 4.1 copies per reaction. Each dilution was analyzed by the EUS qPCR assay with 24 replicates. The 95% limit of detection (LOD_95%_) was determined by calculating the lowest concentration of positive control plasmid DNA that could be detected by the EUS qPCR assay with 95% probability. To confirm the confidence of the LOD value, positive control plasmid DNA at the defined LOD value was analyzed by the EUS qPCR assay with 96 replicates. The number of replicates detected as positive was recorded. A regression line of the C_t_ values from serially diluted positive control plasmid was generated to determine the cut-off value.

#### Interfering substances

An interference study was performed to examine the potential inhibitory effect of several substances (Vitamin C, fucoidan, β-glucan, enrofloxacin, ampicillin, kanamycin, trimethoprim, florfenicol) that might be present in the sample on EUS qPCR assay inhibition. Positive control plasmid DNA (1E+05 copies·mL^-1^) with and without eight substances (S3 Table) and a negative sample with the corresponding substances were analyzed by the EUS qPCR assay in triplicate. The difference in the mean C_t_ value of the positive control plasmid with and without each substance was calculated and expressed as ΔC_t_.

#### Sensitivity comparison between three WOAH-recommended conventional PCR assays and the EUS qPCR assay

The sensitivity of the EUS qPCR assay was compared to that of the three WOAH-recommended conventional PCR assays. DNA was extracted from the mycelia and 2E+06 zoospores of *A*. *invadans* using the DNeasy Power Soil Pro Kit (Qiagen, Germany) according to the manufacturer’s protocol. The DNA concentration of *A*. *invadans* mycelia and zoospores was 47.8 and 6.67 ng·μL^-1^, respectively, measured using a NanoVue Plus spectrophotometer (Biochrom LTD Cambridge, England). The DNA concentration of *A*. *invadans* mycelia was adjusted to a stock solution concentration of 10 ng·μL^-1^. Subsequently, DNA stock solutions from *A*. *invadans* mycelia and zoospores were 10-fold diluted in the elution buffer with and without DNA extracted from healthy gourami tissue (20 ng·μL^-1^). Each dilution (5 μL) was analyzed by the three conventional PCR assays recommended by the WOAH (Table 1) and the EUS qPCR assay and performed in triplicate. However, we were unable to generate PCR products using the two WOAH recommended methods originally developed by Phadee et al. [19] and Vandersea et al. [20]. Hence, in this study, we modified the PCR conditions from a 65 °C annealing temperature and 25 PCR cycles [19] to 59 °C and 35. We also used 0.5 μM of Ainvad-2F/Ainvad-ITSR1 primers instead of 25 pM described by Vandersea et al. [20].

### Precision

#### Dynamic range and efficiency of the assay

A standard curve was constructed by analyzing 10-fold serially diluted positive plasmid DNA ranging from 1E+07 to 1E+01 copies per reaction by the EUS qPCR assay in triplicate. The linearity (R^2^) and efficiency of the standard curve were calculated based on the results derived from each concentration.

#### Repeatability

The stability of the EUS qPCR assay was determined by within-run, between-run, and between-day experiments according to a previous study [21]. Briefly, positive plasmid DNA at three different concentrations, high (1E+06 copies·rxn^-1^), intermediate (1E+04 copies·rxn^-1^), and low (1E+02 copies·rxn^-1^), was used. Each concentration was analyzed in duplicate, with two runs per day over 20 consecutive days. Each run was separated by a minimum of two hours. The variation of the repeatability test was calculated based on the average standard deviation (SD) values of within-run, between-run, and between-day assays and expressed as the coefficient of variation (CV). The CV was calculated as previously described [22] and expressed by the following formula:

$$CV\left(\%\right)=standard\;deviation*\frac{100}{Average\;Ct}$$

#### Reproducibility test

The inter-operator, inter-instrument, and inter-batch reproducibility were determined. Positive control plasmid DNA at three different concentrations, high (1E+06 copies·rxn^-1^), intermediate (1E+04 copies·rxn^-1^), and low (1E+02 copies·rxn^-1^), was used. Each dilution was analyzed in duplicate, with two runs per day over five consecutive days using three different Exicycler^™^ 96 Real-Time Quantitative Thermal Block (Bioneer, Korea) instruments (EXI-05C-0601022, EXI-05C-0601016, and EXI-05M-0802030), and three different inspectors using three different batches of EUS qPCR assays (220116B, 220117B, and 220118B). The inter-operator, inter-instrument, and inter-batch variation in C_t_ values were calculated and expressed as the CV as described above.

#### Whole-system failure rate

The stability of the EUS qPCR assay was determined by analyzing 96 replicates of positive control plasmid DNA at a concentration of two times the defined LOD using the EUS qPCR assay. The percentage of amplification was calculated.

### Statistical analysis

Statistical probit analysis, a non-linear regression model, was performed using commercially available R Studio software [22] to determine the LOD, 95% confidence interval, and cut-off value. The Student’s *t*-test was used to analyze the effect of the potential interfering substances in the EUS qPCR assay using SPSS software version 20.0 (IBM, USA). Statistical significance was considered at *p* < 0.05.

## Results

### EUS quantitative PCR assay design and *in silico* analysis

The target gene chosen was the hypothetical protein encoding gene, which was confirmed to be a single-copy gene using the BLAST program (ftp://ftp.ncbi.nlm.nih.gov/blast/executables/blast+/2.2.28/), as previously described [23]. This gene was only found in the genomes of *A*. *invadans* and *Aphanomyces astaci*. Simulate-PCR analysis showed that EUS-Hypo-F and EUS-Hypo-R primers (Table 1) specifically targeted 80 bp of the hypothetical protein-encoding gene only in *A*. *invadans* genomes and did not amplify any organisms other than *A*. *invadans*. The forward and reverse primers, and probe showed a total of 4, 20, and 15 mismatches and gaps, respectively, in the *Aphanomyces astaci* AP03 sequence (XM_009837853) (S1 Fig). A second set of primers was included in the EUS qPCR assay as internal positive control primers, targeting 121 bp of the movement protein-encoding gene of the TMV particle (Table 1).

### Analytical specificity test

#### Cross-reactivity test

The EUS qPCR assay showed specific amplification using *A*. *invadans* DNA samples, but not other oomycetes, such as *Aphanomyces* species (including *Aphanomyces sp*., *Aphanomyces laevis*, *and Aphanomyces frigidophilus*), and oomycetes isolated from EUS-infected gourami (*Apiotrichum loubieri*, *Antrodiella zonata*, and *Fusarium keratoplasticum*). The developed assay also did not show amplifications using fish-rearing water and Nakdong River water samples containing genomic DNA of other oomycetes, which was confirmed by ITS1/ITS4 primers. Except for BO73/639, non-specific amplification was found with Ainvad-2F/Ainvad-ITSR1 primers using *Aphanomyces frigidophilus* and *Antrodiella zonata*, and ITS11/ITS23 primers using all *Aphanomyces* species, isolated oomycetes, and Nakdong River water tested. No assays showed non-specific priming in fish tissues such as *Cyprinus carpio*, *Colisa lalia*, *Channa argus*, and *Silurus asotus* (Table 2, S2 Fig).

### Analytical sensitivity test

#### Limit of detection and cut-off value

**Fig 1:**
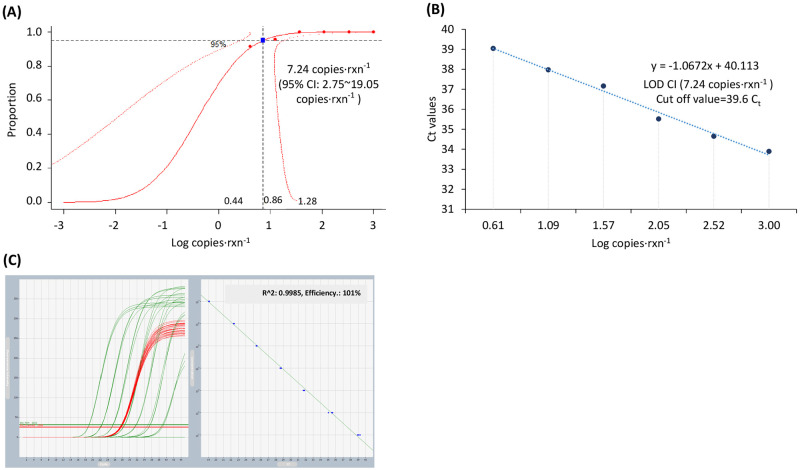
Detection curve for the EUS qPCR assay (A), regression analysis for determining the cut-off value (B), and the dynamic range (C). The solid black line denotes the proportion of detection means with a 95% confidence interval between the solid line in red color.

The sensitivity of the EUS qPCR assay was evaluated using a 3-fold dilution series of positive control plasmid DNA. The LOD_95%_ of the EUS qPCR assay determined by probit regression analysis was 7.24 copies·rxn^-1^ and ranged from 2.75 to 19.05 copies·rxn^-1^ (Fig 1A, S1 Table). Repeating the EUS qPCR assay at 7.24 copies·rxn^-1^ showed a 100% positive rate (S2 Table). A C_t_ value of 39.6 was established as the analytical cut-off limit of the EUS qPCR assay, based on the regression line for the C_t_ distribution for each concentration (Fig 1B). C_t_ values that exceeded this defined limit were considered negative.

#### Interfering substances

No non-specific amplification of interfering substances was seen in negative samples analyzed by the EUS qPCR assay. There was no significant difference in C_t_ values obtained from the EUS qPCR assay using positive control plasmid DNA with and without different substances (*p* > 0.05). The difference in average C_t_ values obtained from the EUS qPCR assay using the positive control plasmid DNA with and without different substances varied from 0.18 to 0.57, with the highest difference of 0.57 C_t_ in the presence of trimethoprim (S3 Table).

#### Sensitivity comparison of three WOAH-recommended conventional PCR assays and the EUS qPCR assay

The sensitivity of three conventional PCR assays, BO73/BO639, Ainvad-2F/Ainvad-ITSR1, and ITS11/ITS23, recommended by the WOAH, and the EUS qPCR assay was assessed using various templates (Table 3). Whereas the three conventional PCR assays showed the same detection thresholds for the mycelia and zoospores of *A*. *invadans* (50 fg and 1 zoospore, respectively), the EUS qPCR assay was 10 times more sensitive (5 fg and 0.1 zoospores, respectively). The EUS qPCR assay showed the same sensitivity using mycelia and zoospores (5 fg and 0.1 zoospores, respectively) with and without fish muscle tissue (Table 3).

**Table 3:** Sensitivity comparisons between WOAH-recommended conventional PCR assays and the EUS qPCR assay using different templates.

Samples	DNA concentration	Percent of positive per 3 replicates (%)			EUS qPCR assay	
		BO73/BO639	Ainvad-2F/Ainvad-ITSR1	ITS11/ITS23	Percent of positive per 3 replicates (%)	C_t_ value (Mean±SD)
Mycelia (fg)	500	100	100	100	100	30.60±0.28
	50	100	100	100	100	33.74±0.79
	5	33	33	33	100	37.75±0.17
	0.5	0	0	0	0	>39.6^2^
Mycelia (fg) +tissue^1^	500	100	100	100	100	30.12±0.23
	50	100	100	100	100	33.58±0.08
	5	33	0	0	100	37.38±0.42
	0.5	0	0	0	33	>39.6^2^
No. of zoospores	10	100	100	100	100	30.70±0.17
	1	100	100	100	100	34.30±0.07
	0.1	0	0	0	100	37.67±0.27
	0.01	0	0	0	0	>39.6^2^
No. of zoospores +Tissue^1^	10	100	100	100	100	30.71±0.40
	1	100	100	100	100	34.56±0.25
	0.1	33	0	0	100	37.76±0.41
	0.01	0	0	0	0	>39.6^2^

^1^Contaning 20 ng·μL^-1^ of healthy gourami (*Colisa lalia*) DNA;

^2^cut-off value of the EUS qPCR assay

### Precision

#### Reaction efficiency

The standard curve was linear over the dynamic range, covering seven orders of magnitude with coefficients of determination (R^2^) of 0.9985 and a reaction efficiency of 101% (Fig 1C).

#### Repeatability

The intra-assay CV determined based on the C_t_ values from 80 separate runs of three different concentrations of positive control plasmid on 20 different days ranged from 0.1 to 0.9%, with the greatest variation of 0.9% observed in the between-day analyses at 1E+06 copies·rxn^-1^ (Table 4).

**Table 4:** Repeatability test summary.

Assay (number)	Category	Positive plasmid DNA (copies·rxn^-1^)		
		High (1E+06)	Intermediate (1E+04)	Low (1E+02)
Within-run (2)	Average C_t_ ± SD	21.41±0.13	27.87±0.14	34.17±0.27
	CV (%)	0.6	0.5	0.8
Between-run (2)	Average C_t_ ± SD	21.41±0.03	27.87±0.03	34.17±0.05
	CV (%)	0.1	0.1	0.2
Between-day (20)	Average C_t_ ± SD	21.41±0.20	27.87±0.19	34.17±0.18
	CV (%)	0.9	0.7	0.5

#### Reproducibility test

The inter-operator, inter-instrument, and inter-batch reproducibility tests showed little variation and ranged from 0.04 to 1.1%. The inter-operator and inter-batch tests showed the greatest variation of 1.1%, observed at 1E+06 copies·rxn^-1^ compared to 0.9 and 0.5% at 1E+04 and 1E+02 copies·rxn^-1^, respectively (Table 5).

**Table 5:** EUS qPCR reproducibility assay.

Assay (number)	Category	Positive plasmid DNA (copies·rxn^-1^)		
		High (1E+06)	Intermediate (1E+04)	Low (1E+02)
Inter-operator (3)	Average C_t_ ± SD	22.15±0.25	28.84±0.25	35.00±0.16
	CV (%)	1.1	0.9	0.5
Inter-Instrument (3)^1^	Average C_t_ ± SD	21.95±0.03	28.50±0.06	34.84±0.01
	CV (%)	0.1	0.2	0.04
Inter-batch (3)^2^	Average C_t_ ± SD	22.16±0.25	28.85±0.25	35.01±0.16
	CV (%)	1.1	0.9	0.5

^1^Exicycler^™^ 96 Real-Time Quantitative Thermal Block (Bioneer, Korea) instruments (EXI-05C-0601022, EXI-05C-0601016, and EXI-05M-0802030);

^2^EUS qPCR assay 220116B, 220117B, and 220118B

#### Whole-system failure rate

The EUS qPCR assay was completely stable, with a positive rate of 100% at twice the LOD value (S4 Table).

## Discussion

Epizootic ulcerative syndrome is considered a global threat to the aquaculture industry due to its ability to expand to new geographical regions and host species [5]. Oidtmann et al. [9] reported that the PCR conditions described by Phadee et al. [19] and Vandersea et al. [20] were unable to generate PCR products using DNA from *A*. *invadans* NJM9701. In this study, the PCR conditions for ITS11/ITS23 and Ainvad-2F/Ainvad-ITSR1 primers also failed to amplify DNA from *A*. *invadans* NJM9701 as described in the Methods section on analytical sensitivity. Thus, the modified conditions described in the Methods section on analytical sensitivity were used and proved to be working. Although conventional PCR assays are recommended by the WOAH, they are usually less sensitive and require more time than qPCR. Also, ITS11/ITS23 primers [19] were reported to have low specificity as they cross-reacted with the genomic DNA of *A*. *frigidophilus* in our study and in a previous study [9]. The detection and quantification of pathogens in water samples have become very important as the field of environmental DNA (eDNA) analysis has rapidly developed over the past decade, and the technique is increasingly used for detecting aquatic pathogens in aquaculture [11, 12]. In this study, we developed a rapid, specific, and sensitive diagnostic assay for the detection and quantification of *A*. *invadans*. In particular, analytical validation, including specificity and sensitivity, was performed, confirming confidence that the assay performs consistently and reliably.

The motile zoospores of *A*. *invadans* attach to the damaged skin of fish, and when the zoospores germinate, their hyphae penetrate the fish skin and invade deep into lower tissues, causing clinical signs of mycotic granulomas in fish skeletal muscle [24]. Hence, the muscle tissue next to or underneath the ulcer is the best tissue for oomycete isolation [25]. Although the WOAH recommends using fish with minor clinical signs, the contamination of samples with other microbes is inevitable. Therefore, the specificity of PCR assays for *A*. *invadans* is essential. The results in this study demonstrated that the EUS qPCR assay was highly specific as no non-specific amplifications occurred in oomycetes isolated from EUS-infected gourami, other closely related *Aphanomyces* species, or river water samples containing the genomic DNA of other oomycetes. However, PCR using the Ainvad-2F/Ainvad-ITSR1 and ITS11/ITS23 primers recommended by the WOAH showed the non-specific amplification of *A*. *frigidophilus* and other oomycete species isolated from EUS-infected gourami (Table 2, S2 Fig). The primers and probe in our EUS qPCR assay targeted 80 bp of the hypothetical protein, which is present in all publicly available *A*. *invadans* genomes (n = 2).

The LOD of the EUS qPCR assay was 7.24 copies per reaction with a cut-off C_t_ value of 39.6. Verification tests performed at 7.24 copies showed a high detection rate of 100% (n = 96/96) (S2 Table), indicating its very high reliability and reproducibility. In general, the use of an IPC aids in the interpretation and ensures the accuracy of the PCR assay as it can help to reduce the likelihood of false negatives, as previously described [26]. Our assay contained an IPC, and the C_t_ values for the IPC under diverse experimental conditions were very constant (mean C_t_ value = 29.2), indicating the high accuracy of the EUS qPCR assay developed in this study. Also, the EUS qPCR assay was 10 times more sensitive than the three WOAH-recommended conventional PCR assays [9, 19, 20] using both mycelia and zoospores of *A*. *invadans*. The same sensitivity was obtained in the EUS qPCR assay when the mycelia and zoospores of *A*. *invadans* (5 fg and 0.1 zoospores, respectively) with and without fish muscle tissue were used. Also, non-significant differences in C_t_ values (*p* > 0.05, S3 Table) were obtained in the EUS qPCR assay when the positive control plasmid DNA with and without eight different substances potentially found in fish and aquaculture systems were used [27, 28]. These results indicated that the presence of excess non-specific DNA did not affect the sensitivity of the EUS qPCR assay.

Repeatability (intra-laboratory) and reproducibility (inter-laboratory) are very important criteria as they can show the ability of an assay to provide consistent results using identical samples within the same or different laboratories, respectively [29]. Repeatability and reproducibility are commonly evaluated based on the CV [30]. Our results demonstrated little variation in the repeatability and reproducibility tests of the EUS qPCR assay, with CV values ranging from 0.1–0.9% and 0.04–1.1%, respectively. The CV values obtained in this study were considerably much lower than the acceptable range of 10%, which is widely used for qPCR assays [31, 32]. In this study, reproducibility tests using three Exicycler^™^ 96 real-time quantitative thermal block instruments and the whole system failure rate were examined, even though they were not commonly performed in other studies. Our EUS qPCR assay showed a minor variability of 0.2% (Table 5) among three PCR instruments and a full stability of 100% (n = 96/96) (S4 Table). The obtained results together with the consistently low variability within and among laboratories, indicated the extremely high consistency, repeatability, and reliability of the developed assay.

## Conclusion

This was the first report on the development of a TaqMan probe-based qPCR assay targeting *A*. *invadans*. Our EUS qPCR assay showed higher sensitivity than the WOAH-recommended conventional PCR assays and provided consistent and reliable results within and between operators. This study also demonstrated that our EUS qPCR assay could detect and quantify *A*. *invadans* not only from infected fish muscle tissue but also water and fecal samples from fish farms and environmental water samples where infected fish exist. This accurate EUS qPCR assay would be of importance in national biosecurity for detecting samples with low *A*. *invadans* levels and biopsy samples collected from asymptomatic or early infected carriers.

## Supporting information

S1 Fig Alignment of the partial hypothetical protein-encoding gene of *Aphanomyces invadans* NJM9701 and NJM0002, and *Aphanomyces astaci* AP03, showing EUS-Hypo-F and EUS-Hypo-R primers and EUS-Hypo-P probe positions. (TIF)

S2 Fig The non-specific amplification of three WOAH-recommended conventional PCR assays using two *Aphanomyces invadans* strains, other oomycetes, four fish species, and water samples. Lane 1, *Aphanomyces invadans* NJM9701; Lane 2, *Aphanomyces invadans* S1; Lane 3, *Aphanomyces sp*. KACC43829; Lane 4, *Aphanomyces laevis* CBS478.1; Lane 5, *Aphanomyces frigidophilus* NJM9665; Lane 6, *Apiotrichum loubieri*; Lane 7, Fusarium *keratoplasticum*; Lane 8, Antrodiella *zonata*; M, 100-bp ladder; Lane 10, common carp (*Cyprinus carpio*); Lane 11, dwarf gourami (*Colisa lalia*); Lane 12, snakehead (*Channa argus*); Lane 13, catfish (*Silurus asotus*); Lane 14, rearing water; Lane 15, Nakdong River water; and lane 16, non-template control. The amplicon size was 564 bp (A), 234 bp (B), 550 bp (C), 800 bp (D), and 2000 bp (E).
(TIF)

S1 Table Determination of the limit of detection of the EUS qPCR assay at six different positive plasmid DNA concentrations. (DOCX)

S2 Table Verification of the limit of detection value using the EUS qPCR assay. (DOCX)

S3 Table Amplification of the EUS qPCR assay using positive control plasmid DNA with and without potential interfering substances. The cycle threshold (C_t_) was expressed as the average ± standard deviation (SD). The ΔC_t_ value is the difference between the C_t_ values of positive samples and positive samples in the presence of substances.
(DOCX)

S4 Table Whole-system failure rate of the EUS qPCR assay. (DOCX)
