# Supplementary figures and images for: Development of a rapid and sensitive real-time diagnostic assay to detect and quantify Aphanomyces invadans, the causative agent of epizootic ulcerative syndrome
Source: PLoS One. 2023 Jun 15;18(6):e0286553. doi: 10.1371/journal.pone.0286553 (PMC10270590; doi:10.1371/journal.pone.0286553)

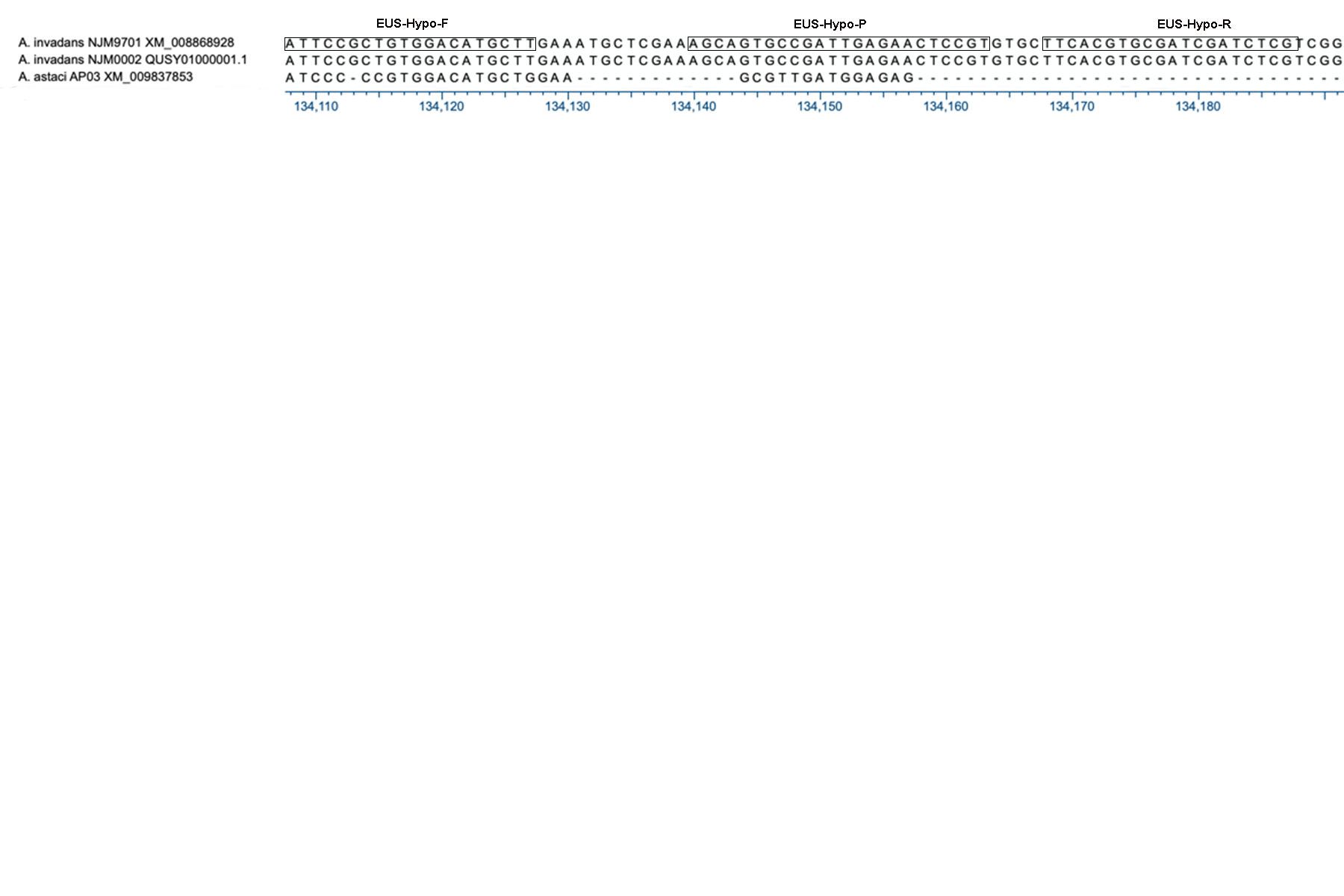

Supplement: S1 Fig — (TIF) [file pone.0286553.s001.tif]

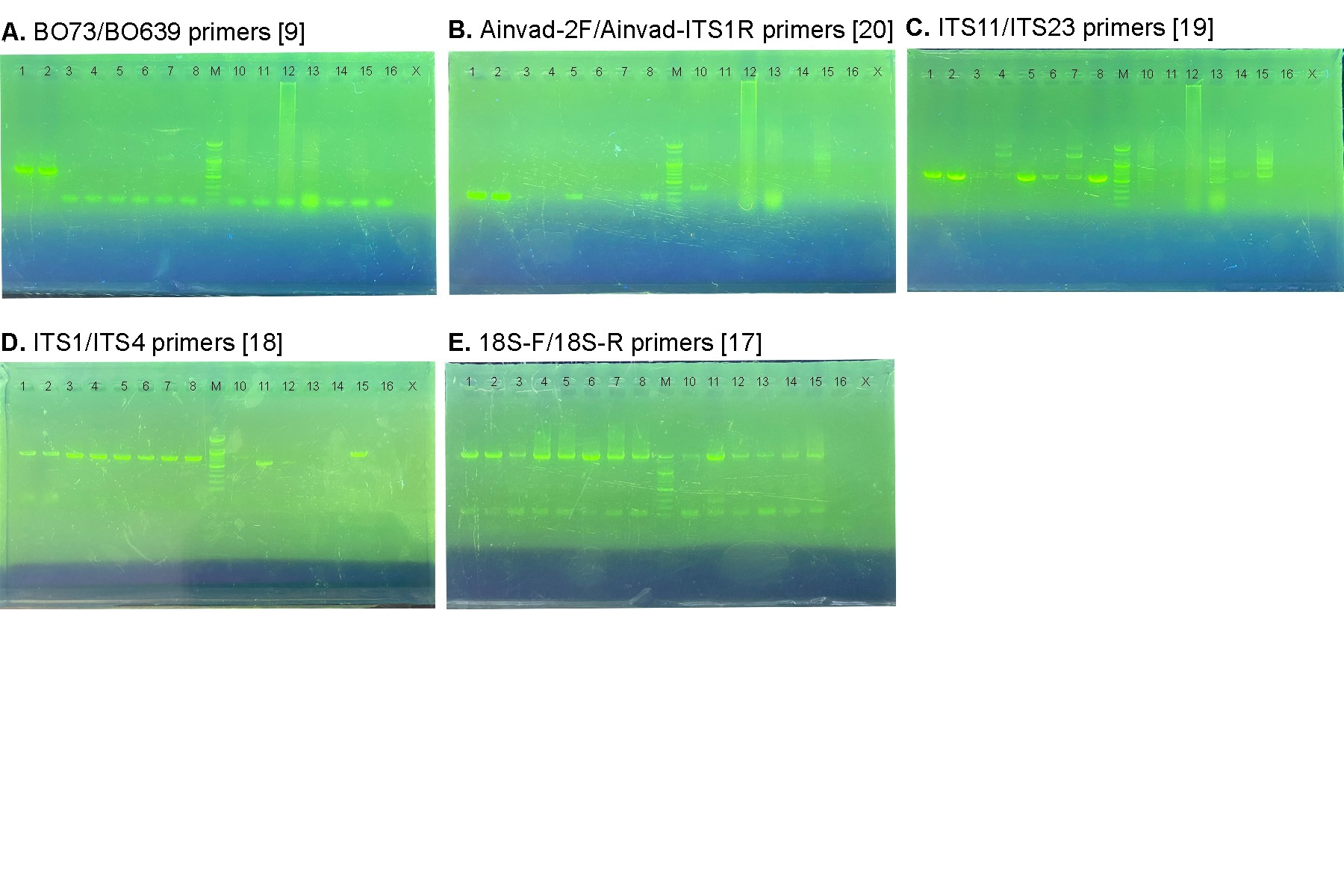

Supplement: S2 Fig — Lane 1, Aphanomyces invadans NJM9701; Lane 2, Aphanomyces invadans S1; Lane 3, Aphanomyces sp. KACC43829; Lane 4, Aphanomyces laevis CBS478.1; Lane 5, Aphanomyces frigidophilus NJM9665; Lane 6, Apiotrichum loubieri; Lane 7, Fusarium keratoplasticum; Lane 8, Antrodiella zonata; M, 100-bp ladder; Lane 10, common carp (Cyprinus carpio); Lane 11, dwarf gourami (Colisa lalia); Lane 12, snakehead (Channa argus); Lane 13, catfish (Silurus asotus); Lane 14, rearing water; Lane 15, Nakdong River water; and lane 16, non-template control. The amplicon size was 564 bp (A), 234 bp (B), 550 bp (C), 800 bp (D), and 2000 bp (E). (TIF) [file pone.0286553.s002.tif]
