# Supplementary material for: Development of a rapid and sensitive real-time diagnostic assay to detect and quantify Aphanomyces invadans, the causative agent of epizootic ulcerative syndrome
Source: PLoS One. 2023 Jun 15;18(6):e0286553. doi: 10.1371/journal.pone.0286553 (PMC10270590; doi:10.1371/journal.pone.0286553)
Supplement: S1 Table — (DOCX) [file pone.0286553.s003.docx]

**S1 Table. Determination of the limit of detection of the EUS qPCR assay at six different positive plasmid DNA concentrations**

| **Replicates** | **Positive plasmid DNA concentrations (copies⋅rnx^--1^)** | | | | | |
| --- | --- | --- | --- | --- | --- | --- |
|  | **1000** | **333** | **111** | **37** | **12.3** | **4.1** |
| 1 | 34.2 | 33.21 | 34.69 | 37.90 | 37.39 | 38.56 |
| 2 | 34.22 | 34.57 | 32.39 | 37.38 | 37.70 | 38.39 |
| 3 | 34.23 | 34.60 | 35.66 | 37.01 | 37.40 | UD |
| 4 | 33.51 | 34.65 | 35.29 | 37.99 | 37.90 | 38.79 |
| 5 | 33.83 | 34.51 | 36.08 | 36.51 | 37.68 | 38.66 |
| 6 | 33.98 | 34.52 | 35.56 | 36.86 | 37.45 | 38.29 |
| 7 | 33.96 | 34.94 | 35.05 | 36.73 | 37.37 | 38.3 |
| 8 | 33.38 | 34.34 | 35.47 | 37.63 | 37.56 | 39.12 |
| 9 | 34.21 | 34.53 | 34.81 | 37.57 | 38.40 | 38.45 |
| 10 | 33.97 | 34.58 | 34.82 | 37.55 | 38.15 | 39.20 |
| 11 | 33.82 | 35.06 | 34.44 | 37.46 | 38.32 | 39.77 |
| 12 | 34.20 | 34.91 | 35.65 | 38.64 | 37.73 | 39.17 |
| 13 | 33.64 | 35.04 | 36.06 | 37.75 | UD | 38.97 |
| 14 | 34.06 | 35.00 | 36.16 | 35.82 | 38.55 | 38.31 |
| 15 | 34.10 | 34.87 | 36.28 | 37.40 | 38.51 | 40.66 |
| 16 | 34.07 | 34.98 | 35.42 | 37.89 | 38.21 | 40.60 |
| 17 | 33.59 | 32.28 | 35.91 | 37.12 | 37.51 | 39.66 |
| 18 | 33.56 | 34.86 | 35.57 | 36.55 | 38.27 | 39.21 |
| 19 | 33.76 | 34.76 | 35.82 | 36.30 | 38.49 | 38.87 |
| 20 | 33.74 | 34.72 | 35.85 | 36.61 | 37.31 | 39.20 |
| 21 | 33.83 | 34.96 | 36.44 | 37.01 | 38.30 | 39.37 |
| 22 | 33.87 | 35.05 | 36.44 | 37.05 | 38.30 | UD |
| 23 | 33.77 | 35.30 | 36.56 | 36.49 | 38.14 | 39.03 |
| 24 | 34.05 | 35.53 | 36.01 | 36.80 | 38.89 | 38.41 |
| **AVG.** | 33.90±0.25 | 34.66±0.66 | 35.52±0.88 | 37.17±0.65 | 39.98±0.47 | 39.05±0.67 |
| **Detection (%)** | 100 (24/24) | 100 (24/24) | 100 (24/24) | 100 (24/24) | 95.83 (23/24) | 91.67 (22/24) |

UD: undetermined
