# Supplementary material for: Development of a rapid and sensitive real-time diagnostic assay to detect and quantify Aphanomyces invadans, the causative agent of epizootic ulcerative syndrome
Source: PLoS One. 2023 Jun 15;18(6):e0286553. doi: 10.1371/journal.pone.0286553 (PMC10270590; doi:10.1371/journal.pone.0286553)
Supplement: S2 Table — (DOCX) [file pone.0286553.s004.docx]

**S2 Table. Verification of the limit of detection value using EUS qPCR assay**

| **No.** | **C_t_ value** | | **No.** | **C_t_ value** | | **No.** | **C_t_ value** | |
| --- | --- | --- | --- | --- | --- | --- | --- | --- |
|  | **EUS** | **IPC** |  | **EUS** | **IPC** |  | **EUS** | **IPC** |
| **1** | 37.35 | 28.34 | **33** | 37.20 | 28.92 | **65** | 34.89 | 30.70 |
| **2** | 37.22 | 28.74 | **34** | 36.90 | 28.95 | **66** | 35.31 | 30.05 |
| **3** | 37.50 | 28.09 | **35** | 37.75 | 28.96 | **67** | 35.84 | 30.10 |
| **4** | 37.00 | 28.46 | **36** | 37.01 | 30.08 | **68** | 36.25 | 29.17 |
| **5** | 36.84 | 27.88 | **37** | 37.75 | 29.62 | **69** | 36.76 | 29.21 |
| **6** | 35.86 | 28.03 | **38** | 36.75 | 29.37 | **70** | 36.49 | 29.75 |
| **7** | 36.38 | 27.82 | **39** | 36.94 | 28.56 | **71** | 36.55 | 29.62 |
| **8** | 37.03 | 28.10 | **40** | 37.05 | 29.88 | **72** | 36.98 | 30.12 |
| **9** | 37.60 | 28.91 | **41** | 36.46 | 28.78 | **73** | 34.51 | 30.07 |
| **10** | 37.20 | 29.26 | **42** | 36.65 | 29.48 | **74** | 35.22 | 30.53 |
| **11** | 36.53 | 28.16 | **43** | 37.29 | 29.99 | **75** | 35.85 | 29.70 |
| **12** | 36.30 | 27.90 | **44** | 36.88 | 30.50 | **76** | 36.08 | 29.10 |
| **13** | 36.29 | 27.98 | **45** | 37.11 | 30.55 | **77** | 36.43 | 28.64 |
| **14** | 36.48 | 27.90 | **46** | 36.98 | 29.05 | **78** | 36.54 | 28.94 |
| **15** | 36.46 | 29.52 | **47** | 37.20 | 28.59 | **79** | 37.97 | 29.69 |
| **16** | 35.84 | 28.71 | **48** | 37.68 | 28.71 | **80** | 37.30 | 28.85 |
| **17** | 37.34 | 28.75 | **49** | 36.26 | 30.46 | **81** | 35.90 | 30.77 |
| **18** | 37.01 | 28.67 | **50** | 36.06 | 28.49 | **82** | 35.42 | 30.45 |
| **19** | 36.55 | 28.04 | **51** | 37.42 | 30.35 | **83** | 35.82 | 29.47 |
| **20** | 36.40 | 27.42 | **52** | 36.60 | 30.23 | **84** | 36.28 | 29.32 |
| **21** | 35.84 | 27.82 | **53** | 37.66 | 30.45 | **85** | 36.62 | 29.40 |
| **22** | 36.58 | 27.85 | **54** | 36.48 | 29.76 | **86** | 36.59 | 29.12 |
| **23** | 36.50 | 28.15 | **55** | 36.57 | 28.49 | **87** | 37.52 | 30.39 |
| **24** | 36.19 | 28.43 | **56** | 36.49 | 29.21 | **88** | 37.84 | 30.94 |
| **25** | 37.58 | 28.87 | **57** | 35.13 | 29.79 | **89** | 34.96 | 29.64 |
| **26** | 37.30 | 29.42 | **58** | 35.47 | 30.34 | **90** | 33.24 | 29.84 |
| **27** | 37.18 | 28.89 | **59** | 36.22 | 30.02 | **91** | 34.76 | 29.69 |
| **28** | 37.15 | 29.03 | **60** | 36.20 | 30.30 | **92** | 35.60 | 28.83 |
| **29** | 36.44 | 28.79 | **61** | 36.33 | 30.08 | **93** | 36.39 | 29.14 |
| **30** | 37.18 | 27.73 | **62** | 36.89 | 29.87 | **94** | 36.47 | 28.80 |
| **31** | 37.90 | 29.35 | **63** | 36.81 | 29.71 | **95** | 37.20 | 29.34 |
| **32** | 36.56 | 28.31 | **64** | 36.91 | 28.96 | **96** | 36.98 | 29.13 |
| **No. of detection: 96/96 Detection (%) 100** | | | | | | | | |
