# Supplementary material for: Development of a rapid and sensitive real-time diagnostic assay to detect and quantify Aphanomyces invadans, the causative agent of epizootic ulcerative syndrome
Source: PLoS One. 2023 Jun 15;18(6):e0286553. doi: 10.1371/journal.pone.0286553 (PMC10270590; doi:10.1371/journal.pone.0286553)
Supplement: S3 Table — The cycle threshold (Ct) was expressed as the average ± standard deviation (SD). The ΔCt value is the difference between the Ct values of positive samples and positive samples in the presence of substances. (DOCX) [file pone.0286553.s005.docx]

**S3 Table. Amplification of the EUS qPCR assay using positive control plasmid DNA with and without potential interfering substances.** The cycle threshold (C_t_) was expressed as the average ± standard deviation (SD). The ΔC_t_ value is the difference between the mean C_t_ values of positive samples and positive samples in the presence of substances

| **Substances** | **Stock conc.** | **C_t_ value (Average ±SD) (Triplicates)** | | | **ΔC_t_** | ***t*-test (*p*)** |
| --- | --- | --- | --- | --- | --- | --- |
|  |  | **Positive sample** | **Positive sample+ substances** | **Substances** |  |  |
| Vitamin C | 100mM | 34.00±0.23 | 33.63±0.33 | UD | 0.37 | 0.158 |
| Fucoidan | 4mg/ml | 34.00±0.23 | 33.46±0.09 | UD | 0.54 | 0.079 |
| β-glucan | 10mg/ml | 34.00±0.23 | 33.82±0.24 | UD | 0.18 | 0.153 |
| Enrofloxacin | 5mg/ml | 34.00±0.23 | 34.18±0.35 | UD | 0.18 | 0.471 |
| Ampicillin | 10mg/ml | 34.00±0.23 | 33.61±0.34 | UD | 0.39 | 0.310 |
| Kanamycin | 2.5mg/ml | 34.00±0.23 | 33.82±0.28 | UD | 0.18 | 0.481 |
| Trimethoprim | 50mg/ml | 34.00±0.23 | 32.43±0.35 | UD | 0.57 | 0.110 |
| Florfenicol | 10mg/ml | 34.00±0.23 | 33.66±0.10 | UD | 0.34 | 0.075 |

UD: Undetermined.
