# Supplementary material for: Development of a rapid and sensitive real-time diagnostic assay to detect and quantify Aphanomyces invadans, the causative agent of epizootic ulcerative syndrome
Source: PLoS One. 2023 Jun 15;18(6):e0286553. doi: 10.1371/journal.pone.0286553 (PMC10270590; doi:10.1371/journal.pone.0286553)
Supplement: S4 Table — (DOCX) [file pone.0286553.s006.docx]

**S4 Table. Whole system failure rate of the EUS qPCR assay**

| **No.** | **C_t_ value** | | **No.** | **C_t_ value** | | **No.** | **C_t_ value** | | **No.** | **C_t_ value** | |
| --- | --- | --- | --- | --- | --- | --- | --- | --- | --- | --- | --- |
|  | **EUS** | **IPC** |  | **EUS** | **IPC** |  | **EUS** | **IPC** |  | **EUS** | **IPC** |
| **1** | 38.02 | 27.59 | **25** | 37.80 | 27.67 | **49** | 37.26 | 27.41 | **73** | 37.35 | 27.63 |
| **2** | 38.89 | 27.41 | **26** | 38.17 | 27.51 | **50** | 37.87 | 27.33 | **74** | 38.15 | 27.59 |
| **3** | 37.34 | 27.55 | **27** | 37.19 | 27.40 | **51** | 37.81 | 27.45 | **75** | 38.17 | 27.47 |
| **4** | 38.07 | 27.44 | **28** | 37.10 | 27.35 | **52** | 37.35 | 27.14 | **76** | 38.19 | 27.32 |
| **5** | 37.06 | 27.05 | **29** | 37.25 | 27.18 | **53** | 37.02 | 27.04 | **77** | 37.16 | 27.34 |
| **6** | 36.97 | 26.90 | **30** | 37.59 | 26.92 | **54** | 37.30 | 26.95 | **78** | 37.07 | 27.29 |
| **7** | 37.39 | 26.76 | **31** | 37.30 | 26.90 | **55** | 36.58 | 26.98 | **79** | 36.83 | 27.19 |
| **8** | 37.24 | 26.58 | **32** | 37.24 | 26.79 | **56** | 37.04 | 26.81 | **80** | 37.93 | 27.06 |
| **9** | 38.05 | 27.57 | **33** | 38.10 | 27.54 | **57** | 37.94 | 27.57 | **81** | 39.10 | 27.68 |
| **10** | 37.89 | 27.43 | **34** | 37.42 | 27.52 | **58** | 37.10 | 27.44 | **82** | 39.03 | 27.49 |
| **11** | 37.53 | 27.38 | **35** | 37.82 | 27.37 | **59** | 37.36 | 27.44 | **83** | 37.52 | 27.60 |
| **12** | 36.92 | 27.40 | **36** | 37.45 | 27.23 | **60** | 37.31 | 27.21 | **84** | 37.10 | 27.46 |
| **13** | 36.89 | 27.25 | **37** | 37.09 | 27.12 | **61** | 36.96 | 27.14 | **85** | 37.39 | 27.38 |
| **14** | 37.11 | 26.86 | **38** | 37.55 | 27.08 | **62** | 36.50 | 27.03 | **86** | 36.82 | 27.33 |
| **15** | 37.13 | 26.89 | **39** | 36.37 | 26.96 | **63** | 37.06 | 26.85 | **87** | 37.22 | 27.28 |
| **16** | 36.82 | 26.79 | **40** | 36.47 | 26.86 | **64** | 36.92 | 26.86 | **88** | 36.54 | 27.22 |
| **17** | 37.61 | 27.62 | **41** | 37.81 | 27.44 | **65** | 38.37 | 27.48 | **89** | 37.74 | 28.10 |
| **18** | 38.12 | 27.55 | **42** | 37.56 | 27.36 | **66** | 36.96 | 27.46 | **90** | 37.75 | 27.72 |
| **19** | 37.39 | 27.47 | **43** | 36.94 | 27.18 | **67** | 37.16 | 27.45 | **91** | 37.20 | 27.66 |
| **20** | 37.33 | 27.48 | **44** | 37.51 | 27.13 | **68** | 37.20 | 27.17 | **92** | 38.17 | 27.52 |
| **21** | 36.70 | 27.15 | **45** | 37.15 | 27.04 | **69** | 36.62 | 27.10 | **93** | 37.16 | 27.55 |
| **22** | 36.38 | 26.90 | **46** | 37.14 | 26.89 | **70** | 37.44 | 27.09 | **94** | 38.02 | 27.51 |
| **23** | 37.88 | 26.91 | **47** | 36.49 | 26.93 | **71** | 37.02 | 27.07 | **95** | 37.27 | 27.36 |
| **24** | 37.22 | 26.87 | **48** | 37.04 | 26.91 | **72** | 38.51 | 26.97 | **96** | 38.29 | 27.35 |
| **Average C_t_: 37.41 Positive rate (%) =100** | | | | | | | | | | | |
